# Ipsilateral and contralateral carotid stenosis contribute to the outcome of reperfusion treatment for ischemic stroke

**DOI:** 10.3389/fneur.2023.1237721

**Published:** 2023-08-10

**Authors:** Giovanna Viticchi, Lorenzo Falsetti, Alice Riva, Silvia Paolucci, Simone Malatini, Emanuele Guerrieri, Marco Bartolini, Mauro Silvestrini

**Affiliations:** ^1^Neurological Clinic, Marche Polytechnic University, Ancona, Italy; ^2^Internal and Subintensive Medicine, Azienda Ospedaliero-Universitaria delle Marche, Ancona, Italy; ^3^Emergency Medicine Residency Program, Marche Polytechnic University, Ancona, Italy

**Keywords:** carotid ultrasound, contralateral carotid stenosis, ipsilateral carotid stenosis, ischemic stroke, reperfusion treatment

## Abstract

**Introduction:**

Ipsilateral and contralateral carotid stenosis (ICS, CCS) influence acute ischemic stroke (AIS) severity and prognosis. Few data are available about their impact on reperfusion therapies efficacy. Aim of this study was to evaluate the impact of ICS and CCS on the effect of intravenous thrombolysis (IT), mechanical thrombectomy (MT) or both and of antiplatelet therapy (AT).

**Methods:**

We enrolled all the consecutive patients admitted for AIS to our stroke unit and submitted to IT, MT, IT+MT, or AT. We established the presence of a significant ICS or CCS (≥70%) by ultrasound examination or brain angio-CT, or MRI. Clinical and instrumental information were collected; delta National Institutes of Health Stroke Scale (NIHSS) from pre-treatment to patients' discharge was employed as the main outcome measure.

**Results:**

In total, 460 subjects were enrolled, 86 with ICS and 38 with CCS. We observed a significant linear trend of delta (NIHSS) between carotid stenosis categories for patients undergoing IT (*p* = 0.011), MT (*p* = 0.046), and MT+IT (*p* = 0.040), but no significant trend among subjects receiving no reperfusion treatments was observed (*p* = 0.174).

**Discussion:**

According to our findings, ICS and CCS negatively influence AIS patients' outcome treated by interventional therapies. ICS might exert an unfavorable effect both by cerebral hypoperfusion and by continuous microembolization toward ischemic area, while CCS is probable involved in reducing the collateral circles effectiveness. The importance of early carotid stenosis detection and treatment should then be reevaluated not only to manage the prevention approaches but also to obtain insights about post-stroke treatment strategies efficacy.

## 1. Introduction

Carotid stenosis is a major risk factor for acute ischemic stroke (AIS). A recent study on more than 3,500 patients showed an estimated rate of ipsilateral carotid-related acute ischemic stroke of 4.7% over 5 years (1). Several studies have shown that patients with significant ipsilateral carotid stenosis (ICS) have a worse outcome and a higher risk of AIS complications (2).

On the other hand, few currently published investigations have attempted to clarify the role of contralateral carotid stenosis (CCS). Higher mortality in AIS patients with a CCS > 50% and an ipsilateral patent carotid artery has been observed (3, 4). The presence of significant CCS in patients with severe ICS was found to be an independent risk factor for acute cerebral vascular impairment with a 3-fold higher risk of TIA or stroke (5).

The prevalence of ICS in AIS varies between 15 and 20% of cases (2), while data on CCS are lacking: some studies have estimated an incidence of 9% (3). Furthermore, few data are available on the different impacts of ICS and CCS on interventional therapies for AIS. According to international guidelines, patients with AIS undergo intravenous thrombolysis (IT), mechanical thrombectomy (MT), or both (the so-called “bridging therapy”) if the inclusion criteria are met. Antiplatelet therapy (AT) is currently suggested for patients with contraindications or who have missed the time or radiological windows for interventional treatment.

The primary aim of this study was to assess the impact of ICS and CCS on the efficacy of different therapeutic approaches for AIS of the cerebral anterior circulation (IT, MT, IT+MT, or AT), as expressed by the functional status of the patients at discharge from a single-center stroke unit. As secondary outcomes, we evaluated the occurrence of the most relevant AIS complications, such as hemorrhagic cerebral infarction and in-hospital death.

## 2. Materials and methods

### 2.1. Materials

We enrolled all the consecutive patients admitted for AIS to the stroke unit of the “Azienda Ospedaliero-Universitaria delle Marche”, Ancona, Italy, in a timeframe ranging from January 1, 2020 to December 31, 2021. Each patient, after an evaluation of the time from symptoms' onset, clinical history, and clinical-radiological characteristics, was submitted to the best possible treatment according to the International Stroke Guidelines. If a patient did not enter the clinical or radiological interventional therapy window, he was treated only with AT. Before treatment, each patient underwent brain computer tomography (CT) or magnetic resonance imaging (MRI) with intra- and extra-cranial vessel studies and perfusion evaluation. After acute treatment, each patient was admitted to the stroke unit of the Neurological Clinic. We evaluated all the patients using the National Institutes of Health Stroke Scale (NIHSS), the most diffused and validated score for AIS, performed from admission in the emergency department (ED) (before any type of treatment) and daily until discharge from the stroke unit. We decided to use the NIHSS to assess AIS patients to obtain an accurate and reliable score for patients with different clinical characteristics and time spent in our stroke unit. Since our aim was to study the short-term effects of interventional therapies on the patients' outcomes, we chose not to employ other scores, such as the modified Rankin Scale, because they explore clinical AIS effects in the longer term.

Each patient was submitted to a neck vessel ultrasound evaluation within the first 24 h after hospitalization. According to ultrasound and radiological evaluation of the extracranial vessels, we assessed the presence of significant ipsilateral or contralateral carotid stenosis (≥70%). First, we submitted all the AIS patients to carotid ultrasound for an initial evaluation of stenosis, occlusions, or plaque characteristics definition. The sonographer was a single, expert neurologist with peculiar expertise in carotid vessel ultrasound. In the presence of significant stenosis, we submitted the patient to the gold standard method for this disease, represented by angio-CT or MRI of the intra- and extra-cranial vessels to obtain a more specific evaluation and a stenosis grade measurement based on ECST criteria.

Inclusion criteria were as follows: (a) diagnosis of AIS as a first cerebrovascular event in the patient's life; (b) AIS due to an impairment of the cerebral anterior circle; and (c) age >18 years.

Exclusion criteria were as follows: (a) the presence of a bilateral significant carotid stenosis (≥70%); (b) the presence of a complete carotid occlusion or tandem occlusion; (c) patients with an extracranial or intracranial vessel dissection; (d) stent placement or carotid endarterectomy (CEA) in the acute phase of stroke; and (e) significant stenosis or occlusion of vertebral, basilar, posterior communicating, or cerebral posterior arteries.

To obtain a more homogeneous sample and reliable information on the impact of carotid stenosis on the efficacy of treatment, we only considered patients who did not undergo stent placement or CEA during hospitalization. We chose to enroll only patients without previous cerebrovascular events to exclude patients undergoing secondary prevention for stroke. These criteria were verified based on both the clinical history and the absence of a previous ischemic event on radiological examinations or with an Alberta Stroke Program Early CT Score (ASPECTS) > 7 (6).

Furthermore, we selected only stroke due to an alteration of the anterior cerebral circulation in order to evaluate only the brain directly vascularized by the carotid system. To exclude possible confounders, we put as an exclusion criterion the absence of significant stenosis or occlusion of the arteries of the posterior circulation.

For each patient, we collected age, sex, in-hospital death, the presence of vascular risk factors (smoke, hypertension, diabetes, and dyslipidemia), the presence of non-valvular atrial fibrillation (NVAF), divided into pre-existing NVAF (pNVAF) and new-onset NVAF (nNVAF), a previous history of acute myocardial infarction (pAMI), and chronic heart failure (CHF). On the definition of a vascular risk factor, we considered both the pre-existing diagnosis and the use of specific treatments (anti-hypertensive, lipid-lowering, and antidiabetic drugs), but we did not explore the indication for the use of antiaggregants. We also evaluated (i) the type of stroke according to Bamford categorization, (ii) the stroke side, (iii) the occurrence of hemorrhagic infarction after AIS, (iv) the presence and the side of carotid stenosis ≥50%, and (v) the type of procedure performed.

The main outcome measure was the Delta (NIHSS), defined as the difference between the NIHSS at admission to the ED (before any type of treatment) and the NIHSS at discharge from the stroke unit.

### 2.2. Compliance with ethical standards

The Ethics Committee of the Marche Region (CERM), Italy, approved the study. All participants and/or caregivers gave their informed written consent to participate and were treated according to the Declaration of Helsinki.

### 2.3. Variable types

We synthesized age and delta (NIHSS) as continuous variables; in-hospital death, hypertension, diabetes, dyslipidemia, pNVAF, pAMI, CHF, nNVAF, and post-procedural hemorrhagic infarction were synthesized as dichotomous variables; smoking attitude (never-smoker, previous smoker, and current smoker), Bamford stroke type (total anterior circulation stroke-TACS, partial anterior circulation stroke-PACS, lacunar stroke-LACS, and posterior circulation stroke-POCS), stroke side (left, right, and bilateral), carotid stenosis (no stenosis, left internal carotid artery, and right internal carotid artery), and procedure type (fibrinolysis, thrombectomy, fibrinolysis + thrombectomy, and no reperfusion procedures) were collected as multiple-level categorical variables. Finally, we created a dichotomous variable regarding procedure type, considering no procedures vs. any procedure type (fibrinolysis and thrombectomy).

### 2.4. Statistical analysis

Continuous variables were tested for normality with the Kolmogorov–Smirnov test. Normally distributed variables were described as mean and standard deviation and compared with a *t*-test (one level) or an ANOVA (multiple levels). Non-normally distributed variables were described as median and interquartile range and compared with the Mann–Whitney *U*-test (one level) or Kruskal–Wallis *H*-test (multiple levels). When comparing multiple-level variables, we also assessed polynomial differences to test a linear trend between levels.

The relationship between variables was tested with Pearson's bivariate test: variables associated with delta (NIHSS) at a level of *p* < 0.05 were selected as covariates for the multivariate model. Finally, we prepared a generalized linear model (GLM) with delta (NIHSS) as an outcome variable, the interaction between procedure type and carotid stenosis type as an independent variable, the Bamford category as a dependent variable, and the covariates obtained by Pearson's analysis. We considered significant differences with a level of *p* < 0.05 in a two-tailed test. Statistical analysis was performed with SPSS 13.0 for Windows Systems.

## 3. Results

We evaluated 523 consecutive patients with AIS. After the exclusion of 63 patients (57 patients were submitted to stent placement or CEA during the hospitalization, 4 had a carotid dissection, and 2 showed a bilateral significant carotid stenosis), a final sample of 460 subjects was obtained. The baseline characteristics of the included patients are shown in Table 1. In particular, we found 86 patients with significant ICS and 38 patients with CCS.

**Table 1:** Baseline characteristics of the sample.

Age (mean ± SD)		**78.53 ± 11.5**
Male sex (*n*, %)		225 (48.9%)
In-hospital death (*n*, %)		24 (5.2%)
Smoke (*n*, %):	No, never	311 (67.6%)
	Previous smoker	70 (15.2%)
	Current smoker	79 (17.2%)
Hypertension (*n*, %)		346 (75.2%)
Diabetes (*n*, %)		117 (25.4%)
Dyslipidemia (*n*, %)		245 (53.3%)
Previous NVAF (*n*, %)		100 (21.7%)
Previous acute myocardial infarction (*n*, %)		106 (23.0%)
Chronic heart failure (*n*, %)		67 (14.6%)
New-onset NVAF (*n*, %)		74 (16.1%)
Bamford classification (*n*, %)	•TACS	•77 (16.7%)
	•PACS	•165 (35.9%)
	•LACS	•90 (19.6%)
	•POCS	•128 (27.8%)
Stroke side (*n*, %)	•Left	•224 (48.7%)
	•Right	•192 (41.7%)
	•Bilateral	•44 (9.5%)
Post-procedural hemorrhagic infarction (*n*, %)		82 (17.8%)
Carotid stenosis (*n*, %)	•No stenosis	•336 (73.0%)
	•Ipsilateral	•86 (18.7%)
	•Contralateral	•38 (8.3%)
Procedure type (*n*, %)	•Fibrinolysis	•133 (28.9%)
	•Thrombectomy	•44 (9.6%)
	•Fibrinolysis + Thrombectomy	•54 (11.7%)
	•None	•229 (49.8%)
Delta (NIHSS) [median, (IQR)]		3 (5)

NVAF, non-valvular atrial fibrillation; IQR, interquartile range; NIHSS, National Institutes of Health Stroke Scale; TACS, total anterior circulation stroke; LACS, lacunar stroke; PACS, partial anterior circulation stroke; POCS, posterior circulation stroke.

We observed that delta (NIHSS) was significantly higher among patients undergoing any procedure [any procedure: 6 (3) vs. no procedure: 1 (2); *p* < 0.0001]. Patients undergoing any revascularization were older (any procedure: 80.55 ± 9.90 vs. no procedure: 76.49 ± 12.61; *p* < 0.0001), with no gender difference (males in any procedure: 45.0% vs. no procedure: 52.4%; *p* = 0.136). Actively treated patients showed more common TACS or PACS when compared to patients not submitted to revascularization procedures (any procedure: 74.4% vs. no procedure: 30.5%; *p* = 0.0001), increased percent of ICS or CCS (any procedure: 35.9% vs. no procedure: 17.9%; *p* < 0.0001), and a more frequent incidence of hemorrhagic infarction during hospitalization (any procedure: 28.5% vs. no procedure: 6.9%; *p* < 0.0001). Performing a procedure was not significantly associated with a lower risk of in-hospital death (any procedure: 6.90% vs. no procedure: 3.40%; *p* = 0.098).

**Figure 1:**
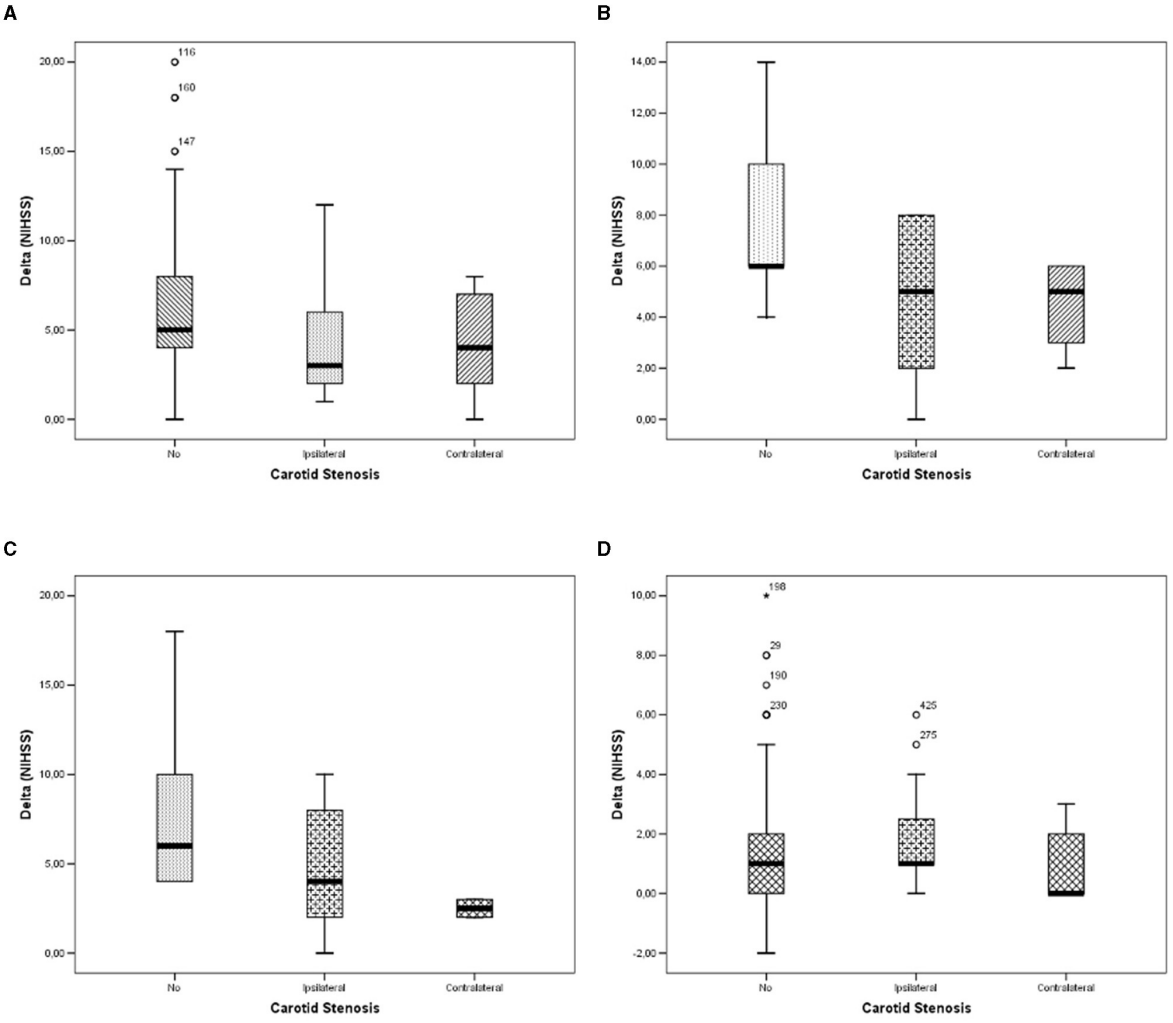
Differences in Delta (NIHSS) between carotid stenosis categories: **(A)** in patients undergoing fibrinolysis; **(B)** in patients undergoing thrombectomy; **(C)** in patients undergoing fibrinolysis and thrombectomy; and **(D)** in patients undergoing no interventional therapies.

We observed a significant linear trend of delta (NIHSS) between carotid stenosis categories for patients undergoing IT (Figure 1A; *p* = 0.011), MT (Figure 1B; *p* = 0.046), and IT+MT (Figure 1C; *p* = 0.040), but we did not observe any significant trend among untreated subjects (Figure 1D; *p* = 0.174). Pairwise comparisons are shown in Table 2. Pearson's bivariate analysis underlined that delta (NIHSS) was significantly associated with sex (*p* = 0.30), Bamford category (*p* = 0.0001), stroke side (*p* = 0.034), hemorrhagic infarction (*p* = 0.0001), in-hospital death (*p* = 0.0001), dyslipidemia (*p* = 0.024), and procedure type (*p* = 0.0001).

**Table 2:** GLM/multivariate model results.

**Source**	**Type III SS**	***df***	**Mean square**	***F***	***P***
Corrected model	3,211.549	21	152.9309	19.81846	0.0001
Intercept	235.7041	1	235.7041	30.54511	0.0001
Procedure	462.5736	3	154.1912	19.98178	0.0001
Carotid stenosis	295.6055	2	147.8028	19.1539	0.0001
Bamford	28.45364	3	9.484548	1.229111	0.299
Age	22.32308	1	22.32308	2.892868	0.090
Sex	16.28369	1	16.28369	2.110219	0.147
Side	8.359958	1	8.359958	1.083375	0.298
Hemorrhage	4.359674	1	4.359674	0.564974	0.452
Dyslipidemia	1.43379	1	1.43379	0.185806	0.667
Death	263.7999	1	263.7999	34.18608	0.0001
Procedure ^*^ Carotid stenosis	145.6153	6	24.26922	3.145071	0.0049
Error	3.379.866	438	7.716589		
Total	13.379	460			
Corrected total	6.591.415	459			

Thus, we prepared a generalized linear model with delta (NIHSS) as a dependent variable, Bamford category carotid stenosis type, procedure type, and their intersection as independent variables, and sex, stroke side, hemorrhagic infarction, in-hospital death, and dyslipidemia as covariates. We also added age to the model to assess whether it could affect the delta (NIHSS); however, we did not observe any significant change in model estimates considering or removing this variable. The GLM/multivariate model resulted significantly (Table 2), thus rejecting the H_0_ hypothesis that the product of the design matrix for the independent variables, the matrix of parameter estimates, and the transpose of the design matrix for the dependent variables was a matrix of zeros.

**Figure 2:**
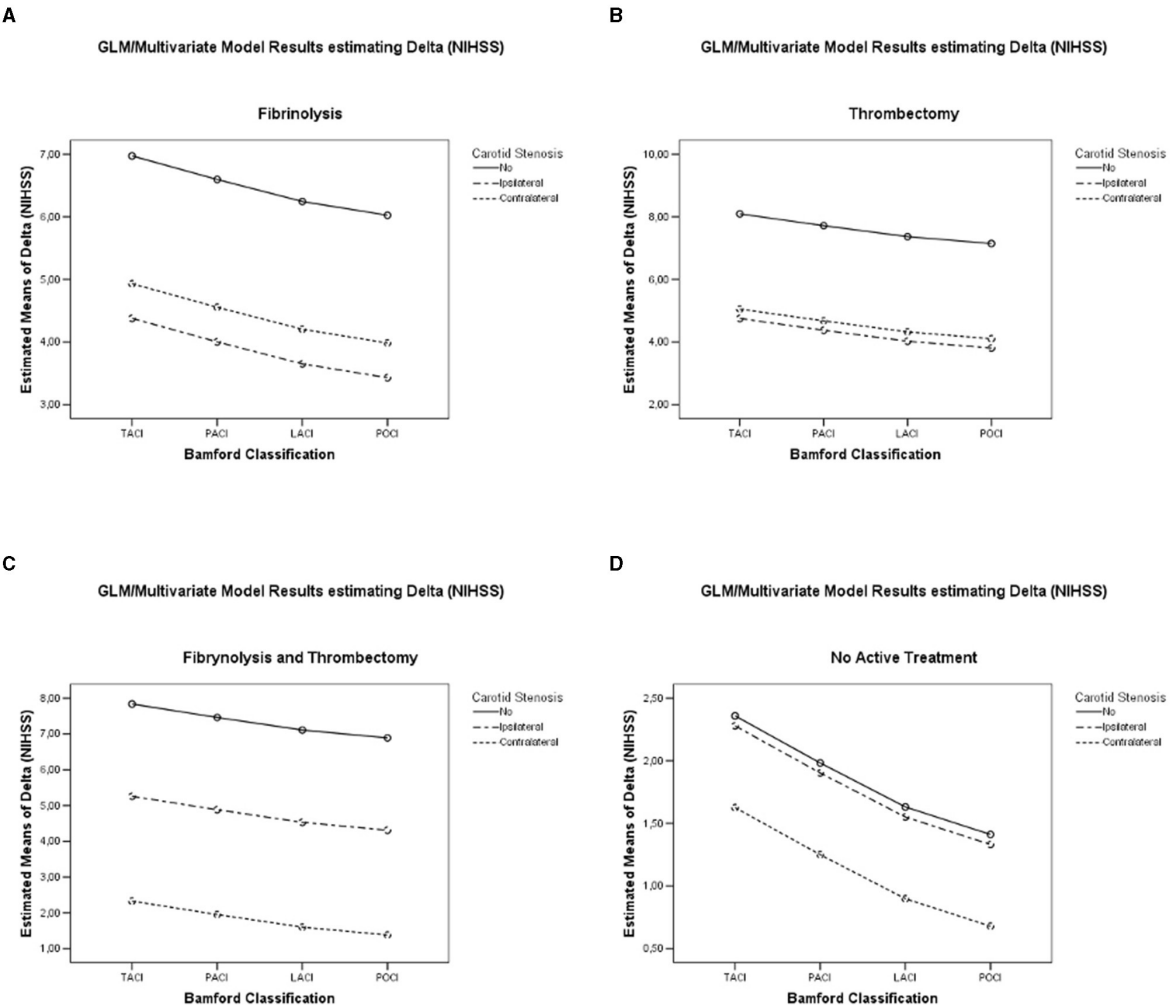
GLM/multivariate results: estimated marginal means of Delta (NIHSS) between carotid stenosis categories and different Bamford categories **(A)** in patients undergoing fibrinolysis; **(B)** in patients undergoing thrombectomy; **(C)** in patients undergoing fibrinolysis and thrombectomy; and **(D)** in patients undergoing no interventional therapies.

Both carotid stenosis (*p* = 0.0001), procedure type (*p* = 0.0001) alone, and their interaction resulted significantly (*p* = 0.0049; Table 2; Figure 2), confirming the observations of the ANOVA model. Among the covariates, in-hospital death and post-procedural hemorrhagic infarction were significantly associated with a significant variation of the dependent variable (Table 2).

## 4. Discussion

### 4.1. Discussion

The percentage of patients with significant carotid artery stenosis in our sample is consistent with data from the literature. Our results confirm that significant carotid stenosis has a significant negative impact on prognosis in patients with AIS. In particular, subjects with ICS presented worse outcomes when treated with IT and MT compared to subjects without significant carotid stenosis. When patients were treated with bridging therapy, the worst outcome was associated with the presence of CCS. If patients did not receive IT or MT, the outcome of subjects with CCS was not significantly different from that of subjects without significant carotid stenosis, while ICS remained a significant risk factor for a worse outcome.

Obviously, the clinical outcome of AIS is predicted by several factors, which are also represented by the patient's age (7), delayed reperfusion treatment (8), clinical severity (9), the proportion of effective reperfusion achieved (8), or comorbidities (9, 10), but carotid stenosis plays a central role in the early and late efficacy of therapies.

Experimental studies have demonstrated a strong relationship between the degree of ipsilateral carotid stenosis and thrombolytic recanalization during AIS, showing a very low rate of recanalization for stenosis > 70% (11). The Interventional Management of Stroke (IMS)-III study showed that patients with an ICS > 70% presented a significantly longer mean time to reperfusion after endovascular treatment, whereas mTICI scores, 90-day mRS scores, or major complications, such as cerebral bleeding rates, did not show a significant difference from the group with non-significant stenosis (12).

CCS is also associated with a worse outcome in AIS. Maus et al. showed an increased rate of unfavorable clinical scores and increased mortality in patients with significant stenosis contralateral to MT-treated cerebral occlusion (3). A CCS is related to a significantly impaired collateral circulation status and is strongly associated with the outcome of AIS (13). A pre-existing significant ICS with a non-significant CCS is associated with improved collateral vessel status, up to four times more likely than in patients without severe ipsilateral carotid stenosis (14).

Our results showed that ICS and CCS have a different impact on the efficacy of treatment, probably because they act through different mechanisms in influencing cerebral perfusion status. ICS may have a direct effect on cerebral hemodynamics with a reduction in cerebral flow in the acute phase of stroke; furthermore, ipsilateral carotid plaques could generate multiple embolisms at different time points in AIS. Transcranial Doppler (TCD) studies showed continuous microembolization from significant carotid stenosis despite antiplatelet therapy (15), and patients with ICS and microemboli at TCD evaluation had a 1-year stroke risk of ~15.6% compared to 1% of those without microemboli (16).

This mechanism is the main cause of a negative outcome. In this respect, several studies have shown that chronic hypoperfusion can be asymptomatic even over a long period of time with good contralateral compensation, increased over time, by collateral flow (5).

ICS plays a role in the compensatory mechanisms of cerebral microcirculation. Chronic carotid stenosis causes increasing stress on the compensation mechanism for hypercapnia, resulting in a chronic impairment in cerebral vasomotor reactivity (CVR). During AIS, when hypoxia and hypercapnia amplify the ischemic damage, the CVR cannot increase any further, resulting in reduced blood perfusion over the ischemic region (17, 18) and a reduction of the penumbra area.

CCS probably plays a preferential role by contributing to collateral circle efficiency, a fundamental protective mechanism activated by the brain during ischemic suffering. Poor collateral circles contribute to a more extensive final infarct, which is directly related to a worse outcome (3, 19). This occurs because the patent carotid artery chronically compensated for the circulation supported by the contralateral carotid artery with significant stenosis, resulting in poor collateral circulation development. During AIS ipsilateral to the patent carotid artery, the collateral circles supplied by the CCS are not ineffective in supporting the contralateral acute occlusion (3, 4).

Ultimately, a carotid stenosis results in maximal chronic utilization of the ipsilateral collateral circles [documented by numerous studies on vasoreactivity ipsilateral to carotid stenosis (17, 18)]. In the case of acute occlusion of a cerebral vessel with an ICS, the ipsilateral collateral circles are unable to increase their action, and the contralateral collateral circles are preferentially activated. Thus, in the case of occlusion and ICS, the contralateral carotid artery-supported collateral circles are effective (but in ICS, there are many other mechanisms, such as hypoperfusion or embolization, that contribute to a negative outcome). On the other hand, CCS results in maximal chronic use of the collateral circles of one's own side of the brain, which are unable to help significantly if an acute occlusion occurs on the other side of the cerebral circulation.

All these considerations regarding cerebral circulation are particularly relevant when AIS is treated with IT or MT. MT is only feasible in the presence of a large vessel occlusion (LVO), and good collateral circulation is considered one of the most important predictors of efficacy (20). It is very effective in LVO occlusions caused by short thrombi or in lacunar strokes, where good cerebral perfusion and self-regulating capacity are supported by valid cerebral small vessel circulation. Our data showed that carotid stenosis may be a negative outcome factor in all types of AIS.

Furthermore, when compared to subjects who did not receive any acute treatment for AIS, patients undergoing IT or MT presented a significantly better outcome, as expressed by a significantly higher Delta (NIHSS). Our data showed that patients undergoing interventional therapies were older and affected by the most severe types of stroke (TACS and PACS) compared to patients treated only with AT, strongly highlighting the efficacy of these approaches. With regard to the main complications of interventional therapies, cerebral hemorrhage occurs more frequently in patients treated with IT or MT. These data are well known in the literature, especially in older patients (7, 21, 22). Despite this, in-hospital deaths are not significantly different between the two groups, confirming the well-known evidence that patients undergoing interventional therapies have an overall better outcome (7, 23) than untreated patients. In our sample, these data are valid for all stroke types according to the Bamford classification.

For our study, we selected all patients who did not undergo early treatment of carotid stenosis (stent placement or CEA). Early treatment of carotid stenosis, even during MT, is a widely discussed topic with conflicting supporting data. The results of our study underline the relevance of carotid stenosis in the outcome of patients with AIS and could support the indication for early treatment. Recent guidelines confirm the indication for the best medical treatment for carotid stenosis before reaching CEA or stent placement, especially in primary prevention (24). Unfortunately, most subjects, even with multiple vascular risk factors, have never performed a carotid ultrasound in their lives and are unaware of the presence of significant carotid stenosis.

The implementation of a rapid, non-invasive, and comprehensive examination in the general population should be strongly encouraged. Data on the low number of complications after CEA or stent placement could be an additional safety factor. A recent meta-analysis showed that carotid stent placement during MT was associated with a better functional outcome compared to patients without stenting but had a higher risk of cerebral hemorrhage (25).

Finally, our study had the scope to support the idea that carotid stenosis is a fundamental risk factor for all stroke types, not only for atherothrombotic ones. ICS determines hypoperfusion, embolization, and chronic impairment in cerebral vasomotor reactivity. CCS induces a progressive derangement of the collateral circles of one's own side of the brain, which could not be effective during an AIS on the other side of the cerebral circulation. Both ICS and CCS had a worsening effect on the efficacy of IT and MT. All these elements give carotid stenosis a pivotal role in the outcome of all strokes.

The recognition of significant carotid stenosis provides the absolute indication to start the best medical therapy, according to all recent guidelines. Furthermore, the best medical therapy may reduce the microembolization rate documented by the TCD examination (26). The best medical therapy is the recommended approach for carotid stenosis to avoid or delay CEA or stent placement. We hope that our data can serve to raise awareness for an early diagnosis of significant carotid stenosis in order to start timely with the best medical approach.

### 4.2. Study limitations

This study has several limitations. First, we only examined patients who did not undergo CEA or stent placement, and we did not have a control group for these patients. Furthermore, we only considered outcomes after the first few days post-AIS. In a future study, we hope to examine outcomes over a longer period to better assess the role of carotid stenosis on residual disability. In particular, in future studies, we aimed to perform a radiological follow-up to better assess both the recanalization and the quality of collateral circles after an acute event. We do not have complete data on plaque characteristics: this information could improve our knowledge of the impact of carotid stenosis in this clinical setting. Another limitation is the lack of accurate information about the possible hypoperfusional condition of the patients, such as a low ejection fraction. We care to specify that this study is a pivotal study on a restricted group of patients in a single center. We hope that it could contribute to highlight the relevant role of carotid stenosis in interventional therapies on all types of strokes and that in the future, multicenter studies will better investigate this hypothesis in a larger population.

## 5. Conclusion

Carotid stenosis plays a central role in the outcome of AIS, with a different role for ICS and CCS. The importance of ultrasound examination for faster and earlier diagnosis seems to be central to the prevention and treatment of AIS and should be suggested, especially in individuals at higher vascular risk.

## Data availability statement

The raw data supporting the conclusions of this article will be made available by the authors, without undue reservation.

## Ethics statement

The studies involving human participants were reviewed and approved by CERM-Comitato Etico Regione Marche. The patients/participants provided their written informed consent to participate in this study.

## Author contributions

GV and LF made a substantial contribution to the concept and design and the interpretation of data and drafted the manuscript. SM and EG made a substantial contribution to the acquisition of data. AR and SP made a substantial contribution to the interpretation of data. MB and MS revised the manuscript critically for important intellectual content and approved the version to be published. All authors contributed to the manuscript revision, read, and approved the submitted version.
